# Analyses of Genetic Variations of Glutathione S-Transferase Mu1 and Theta1 Genes in Bangladeshi Tannery Workers and Healthy Controls

**DOI:** 10.1155/2016/6973057

**Published:** 2016-05-12

**Authors:** Jobaida Akther, Akio Ebihara, Tsutomu Nakagawa, Laila N. Islam, Fumiaki Suzuki, Md. Ismail Hosen, Mahmud Hossain, A. H. M. Nurun Nabi

**Affiliations:** ^1^Department of Biochemistry and Molecular Biology, University of Dhaka, Dhaka 1000, Bangladesh; ^2^Laboratory of Applied Biochemistry, Faculty of Applied Biological Sciences, Gifu University, 1-1 Yanagido, Gifu 501-1193, Japan

## Abstract

Glutathione S-transferases (GSTs) belong to a group of multigene detoxification enzymes, which defend cells against oxidative stress. Tannery workers are at risk of oxidative damage that is usually detoxified by GSTs. This study investigated the genotypic frequencies of GST Mu1 (GSTM1) and GST Theta1 (GSTT1) in Bangladeshi tannery workers and healthy controls followed by their status of oxidative stress and total GST activity. Of the 188 individuals, 50.0% had both GSTM1 and GSTT1 (+/+), 12.2% had GSTM1 (+/−), 31.4% had GSTT1 (−/+) alleles, and 6.4% had null genotypes (−/−) with respect to both GSTM1 and GSTT1 alleles. Among 109 healthy controls, 54.1% were double positive, 9.2% had GSTM1 allele, 32.1% had GSTT1 allele, and 4.6% had null genotypes. Out of 79 tannery workers, 44.3% were +/+, 16.8% were +/−, 30.5% were −/+, and 8.4% were −/−. Though the polymorphic genotypes or allelic variants of GSTM1 and GSTT1 were distributed among the study subjects with different frequencies, the differences between the study groups were not statistically significant. GST activity did not vary significantly between the two groups and also among different genotypes while level of lipid peroxidation was significantly higher in tannery workers compared to controls irrespective of their GST genotypes.

## 1. Introduction

Glutathione transferases (GSTs) protect cells against electrophiles and products of oxidative stress by catalyzing nucleophilic attack of the tripeptide thiol, glutathione on different reactive electrophiles, which otherwise are potentially toxic and can bind to proteins and DNA to cause cellular damage or genetic mutation [1]. These enzymes help to detoxify the compounds, increase their water solubility, and facilitate excretion through urine. In human, a significant number of genetic polymorphisms among the GST enzymes have been described [2]. Individual differences in GST activity are the result of genetic polymorphism of these enzymes. Out of the two GSTs in human, membrane bound GSTs represent 45 of the total proteins present in endoplasmic reticulum and the outer mitochondria while the cytosolic GSTs account for 10% of total cellular proteins [3]. The cytosolic family is further divided into seven classes: Alpha, Mu, Omega, Pi, Sigma, Theta, and Zeta. The Alpha, Mu, and Theta GST classes each contain multiple isoenzymes. One of these genes, GSTM1, encodes for a class *μ* GST isoenzyme involved in polycyclic aromatic hydrocarbons (PAHs) detoxification. Three alleles exist at the locus for human GSTM1, namely, GSTM1^*∗*^A, GSTM1^*∗*^B, and GSTM1^*∗*^0, the latter corresponding to an entire gene deletion [4]. About 50% of the individuals in most Caucasian populations are GSTM1^*∗*^0/GSTM1^*∗*^0 homozygous and lack *μ* GST isoenzyme activity. This has been reported to be possibly associated with an increased susceptibility to different types of cancer including bronchogenic carcinoma [5], urinary bladder cancer [6], and multiple skin tumours [7].

Another polymorphic gene of the same family is GSTT1 which encodes for a class *θ* GST that catalyzes the conjugation of halomethanes in human erythrocytes [8]. Substrates of *θ* GST include industrial chemicals such as methyl chloride, methyl bromide, dichloromethane, ethylene oxide, and diepoxybutane, a reactive metabolite of 1,3-butadiene. Individuals with homozygous GSTT1 gene deletion (GSTT1*∗*0) show a functional deficiency in Theta GST activity in erythrocytes [9]. The prevalence of the GSTT1*∗*0 genotype has been shown to vary between different ethnic groups ranging from 20 to 26% in a group of South Africans to 58% among a group of Chinese people [10, 11]. Some differences have even been found within Caucasian populations [12]. Few reports have linked the homozygous GSTT1*∗*0 genotype to increased cancer susceptibility, but an increased prevalence of the combined GSTM1 and GSTT1 null genotypes has been found among head and neck cancer patients [13]. The GSTT1 null genotype has also been found to influence the age of onset of colon cancer [14].

Workers in the tannery industries directly and inhabitants living around indirectly are at risk of severe health problems due to contamination of drinking water through percolation of untreated or incompletely treated effluents into the ground [15]. Besides, the toxicity can be mediated through inhalation and absorption via skin. Employees of these industries are exposed to higher than normal chromium [16] in the form of either organic chromium or chromium bound to protein (leather dust). Though all organisms have evolved protective mechanisms and programmed responses to limit cellular damage from exposure to toxic compounds in their environment, heavy metals are reported to disturb reactive oxygen neutralizing enzymes and their intoxication causes neurotoxicity, genotoxicity, or carcinogenicity [17]. Gene polymorphism is one of the genetic susceptibility factors that influence the level of DNA damage by affecting the individual's metabolism and repair of various DNA lesions induced by reactive components. For achieving the aim of the present study, allele specific polymerase chain reaction was employed to (i) investigate the distribution of genotypic frequencies of glutathione S-transferase Mu1 (GSTM1) and Theta1 (GSTT1) genes in Bangladeshi tannery workers and healthy controls, (ii) determine the distribution of total GST activity in the plasma of different genotypes among the studied subjects, and (iii) evaluate the level of lipid peroxidation (a marker of oxidative stress) in the plasma of the studied subjects.

## 2. Material and Methods

### 2.1. Rationale of Choosing GSTM1 and GSTT1 out of the Seven Classes of GST Enzymes

Due to the presence of many polymorphisms in GST genes, considerable attention has been bestowed in determining the association of particular allelic variants with the outcome of a variety of diseases. Among all the enzymes of GST family, two major deletion polymorphisms in GSTM1 and GSTT1 genes have shown clinical significance. These deletions result in the absence of enzyme activity, specifically in individuals with null genotypes (absence of both genes due to deletions). The null genotype (GSTM1/GSTT1, −/−) has been associated with altered risk of a variety of pathologies including cancer, cardiovascular disease, respiratory diseases [18–20], and ophthalmologic problems such as cataract [21] and senile macular degeneration [22].

### 2.2. Subject Selection, Sample Collection, and Extraction of Genomic DNA

A total of 190 Bangladeshi people not related to each other were enrolled in this study. Among them, 81 were tannery workers and 109 were healthy control subjects. Before enrolling in the study, each individual was informed about the viewpoint of the study and, after getting their full consent, they were included in the present study and blood samples were collected. Individuals with hypertension and cardiovascular disease related complications, diabetes, chronic kidney disease, or any other major renal diseases and being hepatitis B virus surface antigen (HbsAg) positive were excluded from this study. Healthy control subjects were nontannery workers, randomly selected from the homogeneous ethnic background as of the tannery workers. During interview session, two of the tannery workers were found to be suffering from diabetes and high blood pressure. Thus, finally blood samples from 79 tannery workers were analyzed for the study. All the subjects were also interviewed according to the structured questionnaire and the data were recorded. The study was approved by the Ethical Review Board of the Faculty of Biological Sciences, University of Dhaka, Bangladesh. Five milliliters of blood was collected from each individual in EDTA containing vacutainer tubes by the help of an expert phlebotomist in the presence of the concerned physician. Genomic DNA was extracted and quantity and quality of the extracted DNA were verified according to our previous method [23].

### 2.3. Analyses of GSTM1 and GSTT1 Polymorphisms

GSTM1 and GSTT1 genes were amplified by allele specific polymerase chain reaction (PCR). Primer sequences for GSTM1 were 5′-GAACTCCCTGAAAAGCTAAAGC-3′ (forward primer) and 5′-GTTGGGCTCAAATATACGGTGG-3′ (reverse primer), which produced a 219-base pair PCR product. The GSTT1 primers were 5′-TTCCTTACTGGTCCTCACATCTC-3′ (forward primer) and 5′-TCACCGGATCATGGCCAGCA-3′ (reverse primer), which produced a 480-base pair PCR product. In the same PCR reactions, *β*-globin (268-base pair) was amplified with the primers 5′-CAACTTCATCCACGTTCACC-3′ (forward primer) and 5′-GAAGAGCCAAGGACAGTTAC-3′ (reverse primer) as an internal control of DNA sample. The PCR was performed under the following conditions: 10 minutes of denaturation at 95°C followed by 40 cycles of 1 minute at 95°C, 1 minute at 56°C, 1 minute at 72°C, and a final extension for 10 minutes at 72°C. The amplification products were separated on 2% agarose gels, stained with ethidium bromide for the analyses of genotypes, and only those PCR signals were considered in which the corresponding *β*-globin gene internal control was apparent.

### 2.4. Measurement of the Activity of Plasma Glutathione S-Transferase

Glutathione S-transferase activity was measured according to the method described by Mannervik and Guthenberg [24]. The reaction was carried out in duplicate by observing the conjugation of 1-chloro-2,4-dinitrobenzene (CDNB) with reduced glutathione (GSH). This was done by observing an increase in absorbance at 340 nm. One unit of enzyme will conjugate 10.0 nmol of CDNB with reduced glutathione per minute at 25°C. The results were expressed as U/L.

### 2.5. Determination of the Values of Thiobarbituric Acid Reactive Substances

Thiobarbituric acid reactive substances (TBARS) value was determined according to the method of Yagi [25]. Two mL of working TBA reagent was added to 1 mL sample (100 *μ*L serum + 900 *μ*L saline), followed by the addition of 30 *μ*L of 50 mM butylated hydroxytoluene (BHT). The mixture was incubated for 15 minutes in a boiling water bath. It was then kept in ice bath for 15 minutes. Above steps were repeated for the standards. Then, the sample tubes were centrifuged for 10 minutes at 2000 rpm. Finally, the supernatant was collected to measure the absorbance at 535 nm.

### 2.6. Statistical Analyses

The results were expressed as mean (±SD) and, to compare the differences between different variables from the control and tannery workers, independent Student's *t*-test was performed. A *p* value of less than 0.05 was considered significant. The GSTM1 and GSTTI genotype frequencies were obtained by direct counting. Statistical analyses between different genotypic subgroups of tannery workers and healthy controls were performed using paired sample *t*-tests.

## 3. Results

### 3.1. Anthropometric Data of Healthy Controls and Tannery Workers

The average age and BMI (kg/m^2^) of the total study subjects were 30.95 ± 6.76 years and 23.1 ± 3.21, respectively. The data showed that 54.4% of tannery workers had primary education, 30.4% had secondary education, 12.7% had no education, and only 2.53% had higher education. On the other hand, all the healthy controls were well educated. More than 30% of the total populations were smokers. Their mean systolic blood pressure and diastolic blood pressure were 122.38 ± 13.35 and 79.52 ± 7.7 mmHg, respectively. The mean age of the healthy controls was 28.0 ± 2.0 years. Their average BMI (kg/m^2^) was 24.3 ± 3.34. The mean systolic and diastolic blood pressure of the control subjects were 126.30 ± 15.41 and 81.73 ± 8.80 mmHg, respectively. Twenty percent of them were smokers. A total of 79 workers were included in this study among whom 72 were males and 7 were females. The mean age of tannery workers was 33.90 ± 11.50 years. Their average BMI (kg/m^2^) was 21.90 ± 3.09. The mean systolic and diastolic blood pressure of the tannery workers were 118.46 ± 11.29 and 77.31 ± 6.60 mmHg, respectively. Detailed anthropometric parameters have been presented in Table 1. Among the tannery workers, 40.5% were smokers and 59.5% were nonsmokers. As only 7 female tannery workers participated in this study, the parameters were not shown separately for males and females.

The average duration of work of the tannery workers was 13.65 ± 8.16 years ranging from 5 months to 30 years while their average working hour was 10.6 ± 2.8 hours ranging from 8 to 20 hours. Out of these total tannery workers, 6.33% had pain in the body, 5.1% had cough, 5.1% had breathing problem, 3.8% had headache, and 5.1% of the workers were suffering from weakness. Most of the workers (94.1%) had clear chest sound while 5.1% had noisy sound in their chests. On the other hand, when healthy control subjects were interviewed no such complications were reported.

### 3.2. Frequency Analyses of Polymorphism of Glutathione S-Transferase M1 and Glutathione S-Transferase T1 Genes in Healthy Controls and Tannery Workers

**Figure 1:**
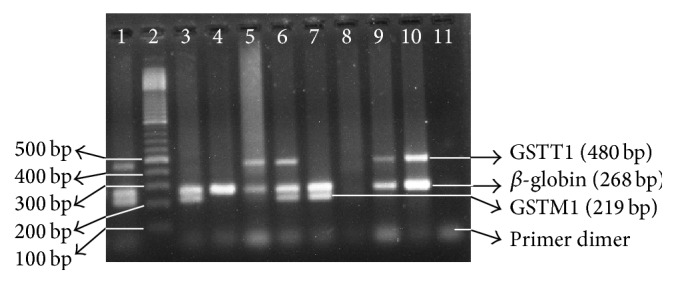
Agarose gel electrophoresis showing the genotypes of GSTM1 and GSTT1 genes. The presence or absence of GSTM1 and GSTT1 genes was detected by the presence of a band at 219 bp (corresponding to GSTM1) and a band at 480 bp (corresponding to GSTT1). The bands at lane 2 indicate molecular marker of 100 bp DNA ladder. A band at 268 bp (corresponding to *β*-globin gene) was always present and was used as internal control to document successful PCR amplification. Lanes 1 and 6 indicate individuals with the presence of both GSTM1 and GSTT1 alleles (+/+). Lane 4 represents an individual with null alleles for both GSTM1 and GSTT1 genes (−/−) showing only one band at 268 bp corresponding to the internal control (*β*-globin gene fragment). Lanes 3 and 7 represent presence of GSTM1 allele and absence of GSTT1 allele (GSTT1 null allele, +/−). Lane 8 is negative control. Lanes 9 and 10 correspond to the individuals with GSTM1 null and GSTT1 present (480 bp) alleles (−/+). The bands at the bottom of the gel are due to the formation of dimer by the primers.

Amplified PCR products were evaluated by agarose gel electrophoresis using a 2% agarose gel and visualized under ultraviolet light after ethidium bromide staining using gel picture analyzer (Alpha Analyzer, US).  Figure 1 represents the GSTM1 and GSTT1 gene polymorphisms by showing different genotypes. *β*-globin gene was used as a positive marker. Sizes of the bands were 219 bp, 268 bp, and 480 bp for GSTM1, *β*-globin, and GSTT1, respectively.

Out of the total study subjects, 94 individuals (50.0%) had both GSTM1 and GSTT1 alleles (+/+) while 23 individuals (12.2%) had GSTM1 allele with null GSTT1 allele (+/−) and 59 individuals (31.4%) had no GSTM1 allele with the presence of GSTT1 allele (−/+). Further, 6.4% (*n* = 12) of the total individuals were null genotypes for both GSTM1 and GSTT1 genes (−/−). Among 109 healthy controls, 54.1% had both GSTM1 and GSTT1 allele, 9.2% had GSTM1 allele with no GSTT1 allele, 32.1% had GSTT1 allele with no allele for GSTM1 gene, and 4.6% individuals represented null genotype. Out of the total 79 tannery workers, 43.3% had both GSTM1 and GSTT1 allele, 16.5% had GSTM1 allele with no allele for GSTT1 gene, 30.4% had GSTT1 allele with no GSTM1 allele, and 8.9% of the total workers represented null genotypes for both GSTM1 and GSTT1 genes. Frequency analyses of the GSTM1 and GSTT1 genotypes have been presented in Table 2.

### 3.3. Glutathione S-Transferase Activity in the Total Participants and Its Genotypic Distribution

**Figure 2:**
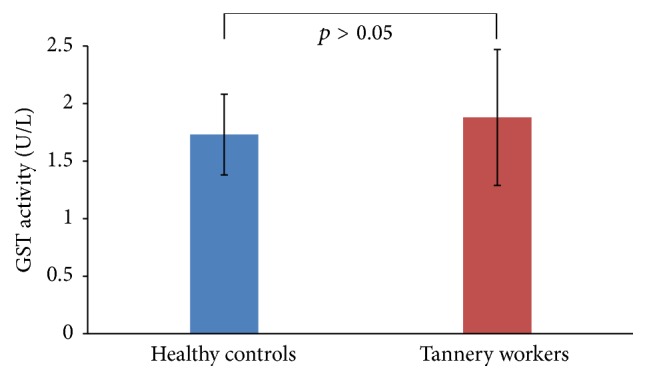
GST activity in the plasma of healthy controls and tannery workers. Though GST activity (U/L) in the plasma of tannery workers was higher compared to that of the control subjects, it did not vary significantly (*p* = 0.071).

The mean activity of GST was estimated to be 1.73 ± 0.35 U/L in the plasma of healthy controls, while this value in the plasma of tannery workers was measured to be 1.88 ± 0.59 U/L. Statistical analysis using independent sample *t*-test revealed that the GST activity between these two groups is not significant (*p* = 0.071) as shown in Figure 2. However, the average GST activities in the smokers and nonsmokers within the tannery workers were 1.92 ± 0.71 and 1.86 ± 0.51, respectively (*p* > 0.05).

**Figure 3:**
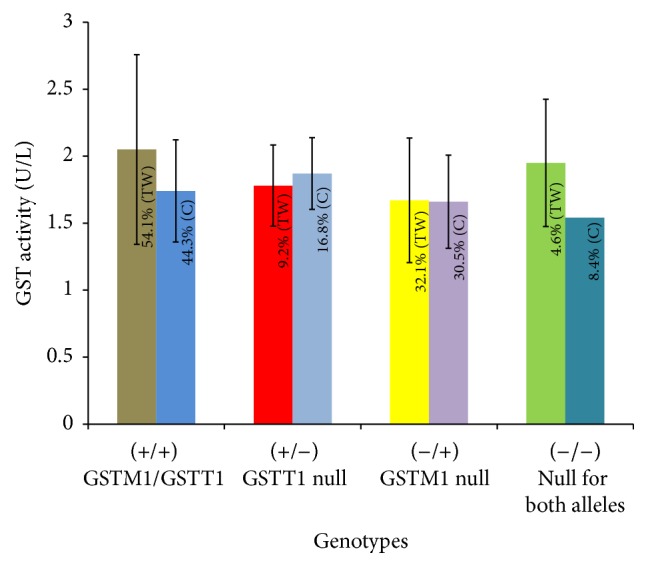
Distribution of GST activity in different genotypes of tannery workers (TW) and healthy controls (C). GST activity varied in different genotypes. The activity was found higher in individuals having both the alleles for GSTM1 and GSTT1 genes. On the other hand, GST activity was found lower in individuals having allele for either GSTM1 or GSTT1 gene (+/−, GSTT1 null genotypes; −/+, GSTTM1 null genotypes). Interestingly, GST activity was also found higher in individuals with null alleles (−/−) for both the GSTM1 and GSTT1 genes. The bars represent the standard deviation of GST activities of each genotype and the GST activities (expressed as U/L) were calculated from the average of two independent experiments for each sample. Values within the bar represent the percentages of respective genotypes in the studied population.

Out of the total study subjects, the mean activity of GST in the individuals with both GSTM1 and GSTT1 alleles was 1.92 ± 0.61 U/L, in individuals having GSTM1 allele with null GSTT1 allele it was 1.81 ± 0.43 U/L, in individuals having GSTT1 allele with null of GSTM1 allele it was 1.66 ± 0.4 U/L, and in individuals with both null genotypes for GSTM1 and GSTT1 genes it was 1.11 ± 0.46 U/L. Tannery workers with both alleles for GSTM1 and GSTT1 genes and double null genotypes (−/−) had higher activity of plasma GST (2.05 ± 0.71 and 1.95 ± 0.48 U/L) compared to their healthy counterparts (1.74 ± 0.38 and 1.54 ± 0.25 U/L, resp.). On the other hand, the plasma GST activities in the individuals either having GSTM1 allele with null allele for GSTT1 gene or having GSTT1 allele with null allele for GSTM1 gene were almost similar to those in the plasma of healthy controls (1.78 ± 0.3 versus 1.87 ± 0.27 U/L and 1.67 ± 0.47 versus 1.67 ± 0.35 U/L, resp.). Statistical analyses revealed that the variations of GST activities in the plasma of tannery workers with different genotypes were not significant. Distributions of GST activities in different genotypic groups of study subjects have been presented in Figure 3.

### 3.4. Levels of TBARS in the Plasma of Total Participants and Their Genotypic Distribution

**Figure 4:**
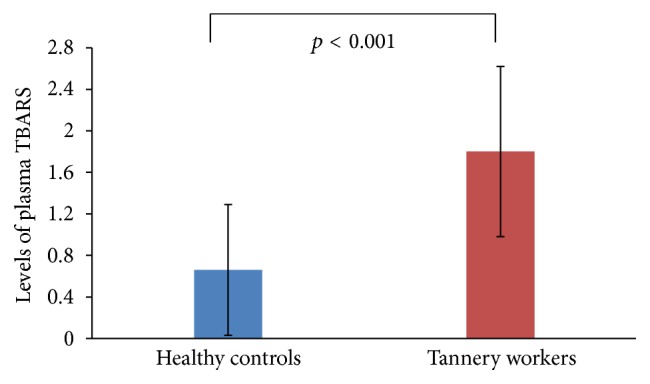
TBARS value in the plasma of healthy controls and tannery workers. Statistical analysis revealed that the level of TBARS was significantly higher (*p* < 0.0001) in tannery workers (1.80 ± 0.82 nmol/mL) than the healthy controls (0.66 ± 0.63 nmol/mL).

The mean level of TBARS in the plasma of the total tannery workers was 1.80 ± 0.82 nmol/mL while in the plasma of the healthy controls it was found to be 0.66 ± 0.63 nmol/mL. The level of TBARS in the plasma of tannery workers was significantly higher than that of the healthy controls as shown in Figure 4.

**Figure 5:**
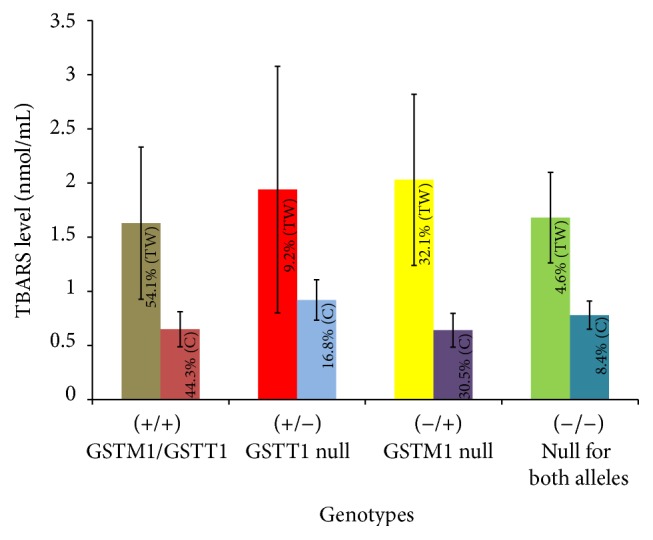
Distribution of TBARS in the plasma of different genotypes of tannery workers (TW) and healthy controls (C). The levels of TBARS in the plasma of all genotypes of tannery workers were found higher than that of the healthy controls. The bars represent the standard deviation of TBARS values of each genotype and the TBARS values (expressed as nmol/mL) were calculated from the average of two independent experiments for each sample. Values within the bar represent the percentages of respective genotypes in the studied population.

Out of the total study subjects, the mean level of TBARS in the plasma of individuals having both the alleles for GSTM1 and GSTT1 genes was 1.63 ± 0.7 nmol/mL while, in case of individuals having GSTM1 alleles with null alleles of GSTT1 gene and in individuals having GSTT1 alleles with null alleles of GSTM1, the mean levels of plasma TBARS were measured to be 1.59 ± 1.16 and 1.33 ± 1.01 nmol/mL, respectively. Tannery workers having both alleles for GSTM1 and GSTT1 genes had statistically significantly higher levels of TBARS (1.63 ± 0.7 nmol/mL) in their plasma (*p* < 0.001) compared to the mean level of the healthy controls (0.65 ± 0.62 nmol/mL). On the other hand, the plasma level of TBARS in the working individuals having GSTM1 alleles with null alleles of GSTT1 gene was 1.59 ± 1.16 nmol/mL, while in their healthy counterparts the mean level was 0.92 ± 0.87 nmol/mL. Also, the level of TBARS in the plasma of tannery workers having only alleles for GSTM1 gene was 2.03 ± 0.8 nmol/mL compared to the level present in their healthy counterparts (0.64 ± 0.56 nmol/mL). It was found that the mean levels of plasma TBARS in these groups of heterozygous tannery workers were significantly higher than those of the control subjects. Moreover, the mean levels of TBARS in the plasma of the tannery workers and healthy controls having null alleles for both GSTM1 and GSTT1 genes were 1.68 ± 0.42 nmol/mL and 0.78 ± 0.08 nmol/mL, respectively (Figure 5).

## 4. Discussion

GSTM1 is one of the most key subclasses of GSTs, which has potent protective role against cancer compared to other GST subtypes [26–29]. Null GSTM1 genotypes have been demonstrated to be most commonly associated with risk of cancers. In addition, McLellan et al. described a rapid GST activity in Saudi Arabian individuals which was attributed to a tandem M1 gene duplication resulting in two functional M1 genes [30] and, thus, it was inferred that this rapid detoxification phenotype could have an increased protective effect against carcinogens. Further, GSTT1 is involved in the bioactivation of carcinogens [31]. Recent evidence has shown that tannery workers are more prone to genotoxicity and increased risk of oxidative stress [32]. Individual variations in GSTT1 and GSTM1 genotypes have been demonstrated to modulate the extent of genotoxicity in tannery workers [33]. This study determined the genotypic distribution of glutathione S-transferase (GST) M1 and T1 genes in Bangladeshi tannery workers and healthy controls along with the status of oxidative stress and total activity of GST enzymes in different genotypes.

Of the total population considered in the present study, half of them were heterozygous with regard to GSTM1 and GSTT1 alleles while only 12.2% were GSTT1 null genotypes and one-third of the population were GSTM1 null genotypes. Similar prevalence of GSTT1 null genotype was reported in Caucasians [34, 35]. Among the world population, the distributions of GSTT1 null genotypes were found to be 47.7% in Caucasians [36], 55–80% in Asians [37, 38], 27% in Eastern and Southern Africans [10], 28–35% in African Americans [26], 22% in Nigerians [27], and 32.4% in Indians [28]. On the other hand, the distribution of GSTT1 null genotypes was found to be lower in our population which has also been reflected in other ethnic groups like 16.7% in Caucasians [36], 24.9% in Blacks [29], and 16.2% in Indians [28]. However, other studies demonstrated that 41% of the Asians have GSTT1 null genotypes [39, 40]. Moreover, the distribution of GSTM1 null genotypes was almost even in the healthy controls and tannery workers as shown in Table 2 which was found to be lower compared to that of the Chinese healthy population reported by Setiawan et al. [41]. Stratification of the genotypes between tannery workers and healthy controls revealed that the null GSTT1 genotypes were higher in tannery workers (16.8%) compared to those of the healthy controls (9.2%). In contrast, the frequency of the GSTT1 gene deletions (null GSTT1 genotypes) in healthy Koreans was almost five times higher [42].

Individuals with homozygous deletions for both GSTM1 and GSTT1 genes (double null genotypes) may contribute to the increased risk for malignancies as a consequence of a decreased efficiency to detoxify possible carcinogens. The occurrences of double null genotypes vary among different ethnic groups. In the present study, the frequency of double null genotype (−/−) was higher in the tannery workers than that of the healthy controls. Considering these facts, though 50% of the total population had both GSTM1 and GSTT1 alleles, rest of them are at risk of genotoxicity and oxidative stress due to the absence of xenobiotics detoxifying GSTM1 and/or GSTT1 alleles.

The GST activity is a parameter for identifying tissue damage and neoplasia [43]. This study further reports varied GST activities in different genotypes. The activity was found higher in individuals having both the alleles for GSTM1 and GSTT1 genes. On the other hand, GST activity was found lower in individuals having either GSTM1 or GSTT1 null genotypes (+/−, −/+) indicating the role of these genotypes in regulating GST activity. Interestingly, GST activity was also found higher in individuals with null alleles for both the GSTM1 and GSTT1 genes. To cope with the oxidative stress, the individuals even with the lowest percentages of double negative alleles (−/−) showed higher GST activity which might be due to expression of other isoenzymes (e.g., GSTP1, GST Alpha, GST delta, GST kappa, GST Omega, and GST Zeta). TBARS are formed as a product of lipid peroxidation due to oxidative stress. Higher TBARS level indicates lower capacity to detoxify reactive oxygen species [44]. It was observed that not only was the TBARS level found to be significantly higher in the plasma of the tannery workers (Figure 4) but also the mean levels of plasma TBARS in all genotypes of tannery workers were higher than those of the healthy controls (as shown in Figure 5). To verify the effect of other factors in regulating GST or TBARS levels we categorized our data according to the smoking status. Although the percentages of smokers were higher in tannery workers compared to those of healthy controls, the average GST activity as well as the levels of TBARS between the smokers and nonsmokers within the tannery workers did not vary significantly. However, due to smaller number of samples, distribution of these values in different GSTM1 and GSTT1 genotypes has not been presented to compare between smokers and nonsmokers.

This study revealed that the genotypic variations with regard to GSTM1 and GSTT1 genes were distributed among the study subjects with different frequencies and there were no significant differences in various genotypes of GSTM1 and GSTT1 gene polymorphisms between tannery workers and healthy controls. GST activity varied among different genotypes while the rate of lipid peroxidation in tannery workers was significantly higher than that of healthy controls irrespective of their different genotypes and this indicated that the tannery workers are at high risk of oxidative damage. Moreover, grouping of tannery workers in different risk groups based on the GST activity (in addition to GSTM1 and GSTT1 genotypes) might enable us to develop a model of regular health screening for the individuals working in this toxic environment. However, more studies with higher number of participants are warranted to establish the frequency distribution of genes for other GST isoenzymes. In summary, only GSTM1 and GSTT1 alleles may not be the sole contributor to GST activity thereby instigating the importance of future studies in this area.

## Figures and Tables

**Table 1:** Anthropometric data of healthy control subjects and tannery workers.

Parameters	Total study subjects (*n* = 129)	Control subjects (*n* = 50)	Tannery workers (*n* = 79)
BMI (kg/m^2^)	23.1 ± 3.21	24.3 ± 3.34	21.9 ± 3.09
	(14.57–31.07)	(14.57–31.07)	(15.7–29.7)
Age (years)	30.95 ± 6.76	28.0 ± 2.0	33.9 ± 11.5
	(15–55)	(19–42)	(15–55)
SBP (mmHg)	122.38 ± 13.35	126.30 ± 15.41	118.46 ± 11.29
	(100–160)	(110–130)	(100–160)
DBP (mmHg)	79.52 ± 7.7	81.73 ± 8.80	77.31 ± 6.60
	(75–90)	(78–88)	(70–90)
Smoking habit (%)	30.25	20	40.5

*n* = number of individuals; numerical values in the parentheses indicate ranges of respective parameters.

**Table 2:** Genotype distribution of GSTM1 and GSTT1 alleles in both healthy controls and tannery workers.

Study subjects	GSTM1/GSTT1	GSTM1/GSTT1	GSTM1/GSTT1	GSTM1/GSTT1
	(+/+), %	(+/−), %	(−/+), %	(−/−), %
Total	50.0	12.2	31.4	6.4
*n* = 188	*n* = 94	*n* = 23	*n* = 59	*n* = 12
Healthy controls	54.1	9.2	32.1	4.6
*n* = 109	*n* = 59	*n* = 10	*n* = 35	*n* = 5
Tannery workers	44.3	16.8	30.5	8.4
*n* = 79	*n* = 35	*n* = 13	*n* = 24	*n* = 7

*n* = number of individuals. +/+ indicates presence of both GSTM1 and GSTT1 alleles; +/− indicates presence of GSTM1 and absence of GSTT1 alleles (GSTT1 null genotypes); −/+ indicates absence of GSTM1 and presence of GSTT1 alleles (GSTM1 null genotypes); −/− indicates absence of both GSTM1 and GSTT1 alleles.
